# What are the kids doing? Exploring young children's activities at home and relations with externally cued executive function and child temperament

**DOI:** 10.1111/desc.13226

**Published:** 2022-01-23

**Authors:** Nicole J. Stucke, Gijsbert Stoet, Sabine Doebel

**Affiliations:** ^1^ Department of Psychology George Mason University Fairfax Virginia USA; ^2^ Department of Psychology University of Essex Colchester C04 3SQ UK

**Keywords:** cognitive control, executive control, executive function, less‐structured time, play, self‐regulation

## Abstract

Young children spend a lot of time at home, yet there is little empirical research on how they spend that time and how it relates to developmental outcomes. Prior research suggests less‐structured time—where children practice making choices and setting goals—may develop *self‐directed* executive function in 6‐year‐olds. But less‐structured time may be related to executive function for other reasons—for example, because it provides opportunities to acquire conceptual knowledge relevant to using executive function on tasks. We thus tested the possibility that less‐structured time is also related to younger children's *externally cued* executive function. In this remote online study, caregivers of 93 3‐ to 5‐year‐olds indicated the amount of time their child was typically spending in various activities while at home during the early phase of the COVID‐19 pandemic. Activities were categorized as *structured* (primarily lessons with specific goals defined by adults or an app), *less‐structured* (wide range of activities permitting choice and interaction with caregiver), *passive* (e.g., watching TV or videos), and *primarily physical* (e.g., bike riding). Children's externally cued executive function was assessed via the Dimensional Change Card Sort (DCCS). Time and variety in less‐structured activities were related to successful switching on the DCCS, controlling for age, family income, caregiver education, and verbal knowledge. Caregivers were more involved in less‐structured versus structured activities. Caregiver ratings of children's temperament were related to how children's time was spent. These findings suggest several new avenues for studying young children's activities at home and their relations with developmental outcomes. A video abstract of this article can be viewed at https://youtu.be/3aGmpSnjuCs

## 1 INTRODUCTION

The quality of a young child's home environment may influence school readiness (e.g., Korucu et al., 2019; Korucu & Schmitt, 2020; Melhuish et al., 2008; Rodriguez & Tamis‐LeMonda, 2011; Rosen et al., 2020; Son & Morrison, 2010), possibly by building *executive function skills*, which involve effectively engaging the capacity to regulate thought and action in the service of goals across contexts. However, research exploring young children's typical activities at home, and links with executive function skills, is scarce. Prior work indicates that time in less‐structured activities—where children have many opportunities to make choices and set goals—predicts 6‐year‐olds’ self‐directed executive function skills (Barker et al., 2014); yet it is not known whether similar patterns hold for younger children.

There is a pressing need to understand young children's activities at home and how they may be related to cognitive skill development, given that children are spending more time at home than ever before as a result of the ongoing COVID‐19 pandemic. At the same time, there is also increasing resistance to structuring young children's time (i.e., lessons and other activities organized by adults with specific goals), with some eschewing structure altogether (Gray, 2013). This resistance has emerged in part as a reaction to the downward extension of formal schooling methods into the preschool years, and in light of beliefs that young children learn best through play and self‐directed exploration (Hirsh‐Pasek et al., 2009; Lillard, 2017). However, all play is not created equal, and empirical exploration of young children's diverse activities at home, and how they relate to development, is needed.

Less‐structured activities have been posited to help children develop *self‐directed* executive function skills—which involve engaging control in response to internal cues—by allowing children to practice using it by making choices (Barker et al., 2014). Self‐directed executive function contrasts with cued executive function, where children engage control in response to others’ explicit instructions. In a previous study, 6‐year‐old children who spent more time in less‐structured activities (e.g., reading, participating in household work, playing non‐physical games) performed better on a measure of self‐directed executive function, the verbal fluency task, where the goal is to maximize the number of words generated in response to a category prompt (e.g., “food”). Optimizing performance on this task requires determining for oneself when to shift to a new subcategory (e.g., from fruit to vegetables). Findings held when controlling for various potential confounds (e.g., socioeconomic status and verbal skills; Barker et al., 2014). The authors theorized that exercising self‐directed control by making one's own choices in daily life fosters its development.

However, time spent in less‐structured activities could be related to executive function for other reasons. For example, by expressing agency, making choices, and spending more time freely interacting with caregivers, children may have more opportunities to acquire knowledge that supports learning to engage control in culturally valued ways (Doebel, 2020). Children who spend time in less‐structured activities like visiting museums, reading books, and exploring ideas in pretend play may acquire conceptual and linguistic knowledge that supports using control on the verbal fluency task (Barker et al., 2014). Moreover, contrary to the notion that caregivers get in the way of children's activities (Gray, 2013), less‐structured time may provide opportunities for children to learn from their caregivers.

**RESEARCH HIGHLIGHTS**

- Explored relation between children's activities at home and externally cued executive function in 93 3‐ to 5‐year‐old children at home at the beginning of the COVID‐19 pandemic
- More time and variety in less‐structured activities was related to externally cued executive function, controlling for age, family income, caregiver education, and verbal knowledge
- Caregivers were more involved in their children's less‐structured versus structured activities
- Caregiver ratings of children's temperament were related to how children's time was spent

**FIGURE 1:**
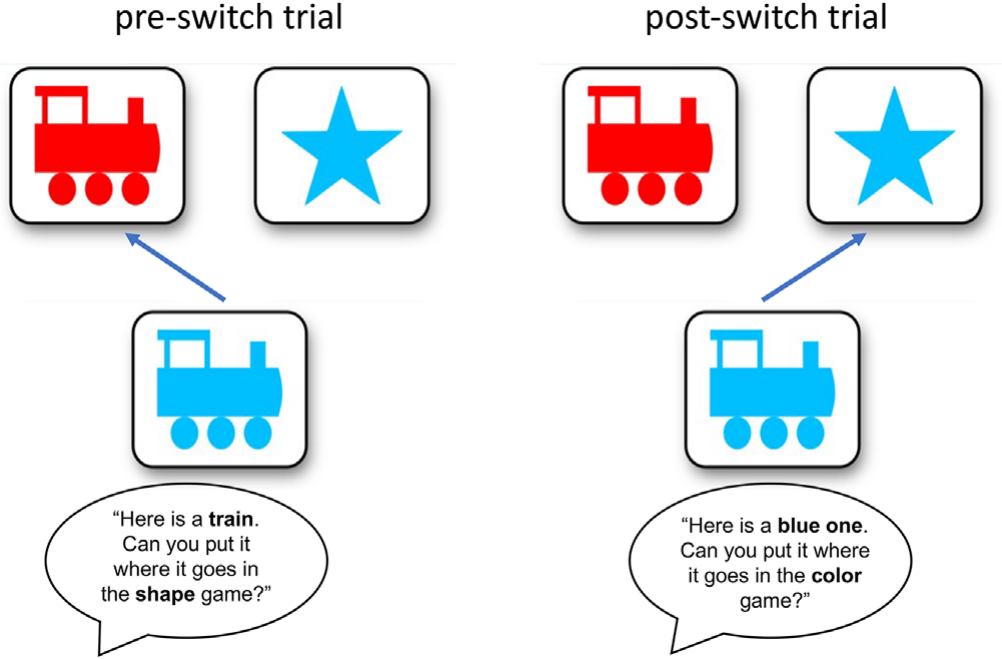
The Dimensional Change Card Sort (DCCS) *Note*. In this task, children match pictures (blue trains, red stars) by one dimension (e.g., shape) during the “pre‐switch phase” and then after several trials are instructed to play a new game (“post‐switch phase”) where they must match the same pictures by another dimension (e.g., color). The figure illustrates what a correct response would look like in each phase.

Such experiences could also support using control on cued executive function measures such as the Dimensional Change Card Sort (DCCS; Figure 1; Zelazo, 2006), which draws on verbal and conceptual knowledge (e.g., knowledge of category labels, dimensions, and contrasting rules; Bardikoff & Sabbagh, 2021; Doebel & Zelazo, 2013; Doebel & Zelazo, 2016; Perone et al., 2015). Consistent with this idea, children who were randomized by lottery to attend a Montessori preschool performed better on measures of externally cued executive function than their conventionally educated peers (Lillard & Else‐Quest, 2006; Lillard et al., 2017). Montessori preschools are characterized by giving children freedom to pursue their interests within a “prepared environment” that includes exposure to a variety of concepts that are relevant to performance on executive function tasks like the verbal fluency task and the DCCS.

On the other hand, the amount of time spent in less‐structured activities could also be related to self‐directed and cued executive function because children who score higher on such measures may have skills in regulating their thinking that makes certain activities more attractive to them and their caregivers.

The current study thus tested the possibility that time spent in a variety of less‐structured activities at home is related to cued executive function skills in younger children as measured by the DCCS. On this task, children are instructed to match picture cards (e.g., red stars and blue trains) by one dimension (e.g., shape) for several trials until they build up a habit, and then are instructed to switch to matching the same cards by another dimension (e.g., color). Many 3‐ and 4‐year‐olds persist in matching by the first dimension despite being regularly reminded of the new rules; yet by five years of age, children typically switch easily (Doebel & Zelazo, 2015). While there is active theoretical debate around how performance on the DCCS should be interpreted (e.g., Doebel, 2020; Perone et al., 2021), the task is an established index of externally cued executive function, with successful performance depending on skills in maintaining and updating representations of task rules, inhibiting representations of no‐longer‐relevant rules, and shifting flexibly to acting on representations of new rules (Zelazo et al., 2013). Accordingly, the DCCS has also been the focus of several theoretical accounts attempting to explain age‐related improvements in performance in an effort to elucidate the mechanisms of executive function development (e.g., Buss & Spencer, 2014; Morton & Munakata, 2002; Zelazo, 2015).

Given that less‐structured activities at home are diverse, we also explored whether the variety of activities was associated with executive function, as children who engage in more diverse activities may have more opportunities to acquire knowledge that can support using executive function. We also explored which specific less‐structured activities tended to be related to performance on the DCCS and how involved caregivers tended to be in less‐structured versus structured activities.

Our second hypothesis was that time spent in *structured* activities (primarily lessons with specific goals defined by adults or an app, as in Barker et al., 2014) would also be associated with performance on the DCCS. While previous research suggests time in structured activities does not relate to self‐directed executive function (Barker et al., 2014), we expected it might be positively related to externally cued executive function as indexed by the DCCS, given that younger children engaging in these activities might experience quality interactions with caregivers that foster knowledge acquisition. However, this relation might be weaker than the relation between less‐structured time and DCCS performance if having more room to express agency and choice is crucial to acquiring knowledge at this age, as has been suggested (e.g., Hirsh‐Pasek, et al., 2009; Lillard, 2017). On the other hand, in some cultural groups (e.g., Chinese American), children experience a fair amount of structure and guidance from adults (Ng & Wei, 2020), which could provide knowledge that supports externally cued executive function. Consistent with these ideas, prior work has found that preschool curricula that introduce more structure have found improvements in children's self‐regulation (Rege et al., 2019). While Barker et al. (2014) did not find a statistically significant relationship between structured time and externally cued executive function (*p* = 0.06), it is possible that there was a relation, but the study was underpowered to detect it given their sample size and measure of externally cued executive function (the Flanker task).

Our third hypothesis was that time engaged with passive media would be negatively associated with performance on the DCCS, consistent with prior work (e.g., Lillard et al., 2015). We analyzed time spent using passive media and engaging in primarily physical activity as additional categories of time use (apart from other less‐structured activities and structured activities/lessons), both to describe the prevalence of these types of activities and to explore their relations with executive function. While we did not formulate a specific hypothesis about the relation between physical activity and executive function, given conflicting findings in the literature (Becker et al., 2014; Cook et al., 2019; Nieto‐López et al., 2020), we nevertheless explored this relation.

Finally, we were also interested in exploring how children themselves might shape how their time is organized by caregivers. For example, caregivers’ perceptions of their child's temperament may influence the opportunities children have to engage in less‐structured activities. Thus, in the reported study, caregivers were asked about how much time they were engaged in the activities with their child and were also asked to report their perceptions of their child's temperament using the Child Behavior Questionnaire (Putnam & Rothbart, 2006) so that we could learn more about how caregivers’ perceptions of their children's dispositions related to how they spent their time home.

## 2 METHOD

The three study hypotheses and associated analysis plan were preregistered on the Open Science Framework: https://osf.io/3k49g/. By preregistering these predictions and being explicit about our exclusion criteria, we aimed to increase confidence that the confirmatory findings are not false positives. Given that these were independent hypotheses and analyses, we did not plan to adjust our alpha level of 0.05 (Lakens, 2016; Rubin, 2021). However, we also report many exploratory findings that the preregistration does not bear on, but that can provide new directions for future confirmatory research.

### 2.1 Participants

Ninety‐three children participated in our study: 33 three‐year‐olds, 31 four‐year‐olds, and 29 five‐year‐olds (*M* = 4.42 years, *SD* = 0.87; range = 3.00–5.92; *n* = 43 female). Participating caregivers were mothers (*n* = 81) and fathers (*n* = 12).

We chose our sample size based on statistical power and practical considerations. To detect a small‐to‐medium effect (*r *= 0.3) with 0.80 power and an alpha level of 0.05 we required at least 83 participants; however, we initially aimed to recruit a larger sample given our plans for exploratory analyses. Our original planned sample size was deemed unfeasible given persistently low recruitment after the first two months, despite ongoing efforts. We began collecting data on May 20, 2020 and decided on July 8th, 2020 (after collecting 85 participants over 2 months) that we would cease collecting data on July 29th, 2020. We recruited eight more children during that period. Prior to stopping data collection no data had been downloaded, reviewed, or analyzed.

Families were recruited from a database of parents and children who had previously indicated interest in participating in developmental psychology research, social media platforms, as well as local school systems, childcare facilities, and community groups. Caregivers answered questions prior to being enrolled in the study to confirm their child did not have any conditions that would interfere with their completion of the tasks, including developmental delays or disabilities and vision and hearing loss. Caregivers were also asked to confirm that they could read English in order to complete the surveys and to confirm that their children understood English in order to follow task instructions. All caregivers provided informed consent in accordance with policies of the Institutional Review Board at George Mason University. Caregivers provided informed consent online when they arrived at the study webpage by clicking a button beside the statement “I agree to participate in this study.”

The racial breakdown of our sample was 76% White, 10% East Asian, 2% Black or African American, 1% South Asian, and 11% multiracial. Five percent identified as Hispanic/Latinx. Most of the responding caregivers completed graduate education (46% had a master's degree and 16% had a Ph.D.). Thirty‐two percent had a bachelor's degree and 3% undertook but did not complete a graduate degree. One percent undertook but did not complete college. Family income was higher than the national average: 31% reported an income of $200,000 or more; 17% reported $125,000‐149,999; 16% reported $150,000‐174,999; 10% reported $100,000‐124,999; 10% reported $75,000‐99,999; 8% reported $175,000‐199,999; and 1% reported $50,000‐74,999.

A majority of caregivers reported that their child was at home in their care (94%), while a small minority reported their child being in the care of a sitter, nanny, or family member (6%). In sixty‐three percent of families, primary care for the child while they were at home was shared between two parents. In the remaining families, primary care was undertaken by mothers (31%), fathers (2%), or a nanny, sitter, or relative (3%). Participating families reported living in the United States (98%) and the UK (2%). Prior to the pandemic, most children (75%) attended some form of school on a part‐ or full‐time basis (preschool, kindergarten, or primary school). The remaining children attended a local or family daycare (15%) or were cared for at home by a parent or relative (4%). Few children were reported as having no formal care (2%). A small proportion of responders left this field blank (4%).

At the time of the study, participating children had been at home for an average duration of 87 days (SD = 19.73 days; range = 58‐142 days), the vast majority being home due to some degree of pandemic‐related restrictions, with 95.7% experiencing some restrictions/partial lockdown and 3.23% experiencing full lockdown.

### 2.2 Procedure

Children's executive function was measured using two online behavioral measures (only one of which provided useable data, as detailed below). These tasks were hosted on PsyToolkit, an online behavioral data collection platform (Stoet, 2010; 2017). Caregivers first completed an online survey in Qualtrics and then were provided with a link to complete the child behavioral tasks.

### 2.3 Measures

Caregivers answered questions in Qualtrics about their child's time use, temperament, and verbal knowledge. These measures are described below.

#### 2.3.1 Children's time use

Caregivers were asked to indicate the amount of time on a typical day, in half‐hour increments, that their child engaged in 32 total activities. This measure was designed to capture children's typical time use across a broad range of activities. We expected that children did not necessarily engage in all reported activities every single day, so we did not require that caregivers ensure that time estimates correspond perfectly to the available hours in a single day. The specific prompt caregivers received was:

Please indicate the amount of time your child is typically spending on each of the following activities during COVID‐19. Time estimates do not need to add up exactly to a full day, but please try to count a specific activity in one category only (e.g., count watching 1 hour of media as either “watching television” OR “watching YouTube videos/DVD or other video media,” but do not count it twice).

We inquired about a wide range of activities, including but not limited to: time spent engaged in arts and crafts, playing with toys, watching TV, pretending, writing, scribbling or doodling, unplanned activities with numbers, and planned writing or musical lessons. Caregivers were instructed to minimize overlap in how they classified an activity (e.g., not including their estimate of time spent playing with toys in their estimate of time spent engaged in pretending).

**Coding of Time Use**. We based our coding of less‐structured and structured time on Barker et al. (2014), with some intentional changes. We initially categorized all activities as less‐structured or structured, following Barker et al. (2014). This coding scheme can be found here: https://osf.io/3k49g/. Barker et al.’s classification was based on previous literature drawing conceptual distinctions between activities involving more‐ or less‐structure (e.g., Fletcher et al., 2003; Larson & Verma, 1999; Meeks & Mauldin, 1990). Specifically, structured activities are typically organized by adult leaders and involve imposed rules, standards, and goals that guide children's activity. Less‐structured activities, on the other hand, such as play, arise more spontaneously and are not directed in this way by adults. In our study, because children were at home, structured activities were primarily lessons or guided activities on computer or tablet apps. Less‐structured activities included reading, playing with toys, engaging in pretense, being involved in cooking or housework, and more. In our preregistration, we departed from Barker et al. by separating passive activities (e.g., watching TV and videos and playing video games) and primarily physical activities (e.g., rough and tumble play, riding a bike) from less‐structured activities to explore their prevalence and relations with externally cued executive function separately. Caregivers were also given the option of specifying up to three additional activities that their child engaged in on a typical day. Authors SD and NJS reviewed these responses independently, agreeing on how they could be recoded and added to one of the existing 26 activity categories. For example, if a caregiver indicated that their child spent an hour “building Lego sets with instructions,” this time was added to the reported time spent “using combinable objects.” We note that our results do not change if we do not integrate these additional text responses.

#### 2.3.2 Caregiver involvement

Caregivers were also asked, for each activity they indicated their child engaged in, to further indicate how much time they were actively involved in the activity with the child. For ease of survey completion, caregivers selected from the following options: 0%, 25%, 50%, 75%, or 100%.

#### 2.3.3 Child temperament

Caregivers completed the Children's Behavior Questionnaire–Very Short Form (CBQ–VSF; Putnam & Rothbart, 2006). This questionnaire asked caregivers to rate how true or false a specific statement was of their child using a seven‐point Likert scale ranging from “extremely true” to “extremely untrue.” The questionnaire included statements about children's behavior in various situations and contexts (e.g., “My child prefers quiet activities to active games,” “My child's feelings are not easily hurt by criticism,” “My child is full of energy, even in the evening”). Scores for three subscales (effortful control, negative affect, and surgency) were computed. These subscales are reliable, with alphas for surgency, negative affect, and effortful control reported as 0.75, 0.72, and 0.74, respectively (Putnam & Rothbart, 2006).

#### 2.3.4 Child verbal skills

We used the Developmental Vocabulary Assessment for Parents (DVAP; Libertus et al., 2015) to provide an index of children's verbal knowledge. Caregivers were shown a list of words and asked to mark words that they have heard their child say. The list included words that children typically learn between ages 2 and 18 years. Scores were computed from the total number of words marked.

#### 2.3.5 Children's executive function

Children completed two executive function measures: the DCCS (Zelazo, 2006) and the Go/No‐Go task (Wiebe et al., 2012). However, the Go/No‐Go task did not yield valid data. Specifically, 33% (29 of 89 who completed this task) of children did not meet the criteria for data inclusion laid out in our preregistration. Children needed to respond accurately to 75% of “go” trials, a basic requirement for establishing a prepotent response that they must attempt to override during “no go” trials. This may have been because the task parameters (following Wiebe et al., 2012) were not optimized for our sample (e.g., duration of stimulus presentation). Thus, the task is not described further. In our preregistration, we stated that we would also ask caregivers to complete a questionnaire measure of executive function, but we ultimately decided not to include the measure in order to reduce the length of the study.

**Dimensional Change Card Sort**. Our online version of the DCCS was created and hosted via PsyToolkit. The task can be found here: https://www.psytoolkit.org/experiment‐library/dccs.html. We aimed to create an online task that provided similar verbal prompts that would occur during an administration of the task in the lab, both to help children understand the task and to stay engaged. We also included special instructions to minimize the likelihood of caregiver interference, detailed below. Finally, we included checks to assess and account for the possibility of caregiver interference. The goal of the task was to match a colored shape (red star or blue train), presented on the bottom center of the screen, to one of two colored shapes presented on the top left and right of the screen (red train and blue star, respectively; see Figure 1).

***Task introduction***. Children and caregivers were first introduced to the task with narration accompanying brief animated instructions. Specifically, they were told that the child was going to play a matching game using the “A” and “L” keys on the keyboard. Children and caregivers were given the option to have the child press the keys themselves, keeping their fingers on the “A” and “L” keys, or to have the child point, with the caregiver pressing the corresponding keys. Caregivers were instructed by the narrator to refrain from correcting the child's responses or giving them any feedback aside from gentle encouragement to pay attention and keep going.

***Key practice***. Next, children were told they were going to practice using the “A” and “L” keys. Children saw two identical gray‐scale smiley faces presented on the top left and right of the screen. An identical gray‐scale smiley face appeared on the bottom center of the screen, and the narrating voice said, “First, let's practice using the keys we're using in our game. Can you press the 'A' key?” If the child did not respond after a 10,000 ms delay or responded incorrectly (e.g., pressed the “L” key instead of the “A” key), the voice said, “Uh oh, remember, you have to press the 'A' key on your keyboard. Let's try again.” The narrator repeated this prompt a maximum of 10 times before moving on or until the correct key was pressed, at which point the narrator said, “That's right, that's the 'A' key.” The same procedure was repeated for the “L” key.

***Pre‐switch Practice Trials***. Next, children were presented with a red train and a blue star in the top left and right of the screen, respectively. The narrator said, “We're going to play a fun matching game. It's called the shape game! In the shape game, all the trains go here, and all the stars go here.” The red train and blue star jiggled when referenced by the narrator. Next, the narrator said, “Here's a train, it goes here in the shape game.” At this point, a blue train appeared in the bottom center of the screen before moving toward the red train in the top left corner and disappearing behind the red train. The narrator then said, “And here's a star, it goes here in the shape game.” Likewise, a red star appears in the bottom center of the screen before moving toward the blue star in the top right corner and disappearing behind the blue star. The narrator concluded by saying, “Now you try.” Children were presented with a blue train in the bottom center of the screen, and asked, “Here is a train. Can you put it where it goes in the shape game?” The blue train remained on the screen for 10,000 ms or until the child pressed the “A” or “L” key. If the child pressed the incorrect key, the narrator said, “Uh oh, nope. The train goes here in the shape game. Let's try another one.” If the child did not respond after a 10,000 ms delay, the narrator said, “Let's try another one,” before presenting the child with another colored shape for the child to match. Children were given a maximum of 10 trials or until they correctly matched each shape, at which point the narrator said, “Yay, that's where the train (or star) goes in the shape game.”

***Pre‐switch Trials***. To introduce the pre‐switch trials, the narrator said, “Okay, now we're going to play for real. Go as fast as you can and try not to make any mistakes.” The instructions related to speed are similar to other validated computerized versions of DCCS (e.g., Carlson & Zelazo, 2014; Zelazo et al., 2013). As in the Toolbox version, for example, children are given ample time to respond (10,000 ms). Also like the Toolbox version, this task was designed to allow other researchers to use it and collect RT data if so desired. We did not plan to analyze RT data because we permitted parents to assist with key pressing. During each trial, the colored shape was verbally labeled (“Here's a train (or star)”). When the child responded, they heard a pleasant game sound. No informative feedback was provided to the child following any pre‐switch trial. There were 12 pre‐switch trials in total presented in a pseudorandom order with the restriction that the same shape could not be presented more than two times in a row.

***Post‐switch Trials***. Upon completing the pre‐switch trials, children were presented with a happy face and the narrator said, “Okay, now we're going to play a new game. We're not going to play the shape game anymore, no way! We're going to play the color game. The color game is different. In the color game, all the red ones go here (the red train in the top left corner wiggles for emphasis), and all the blue ones go here (the blue star in the top right corner wiggles for emphasis). Red ones go here, and blue ones go here. Okay, let's play!” There were 12 post‐switch trials in total, presented in a pseudorandom order with the restriction that a shape of the same color could not be presented more than two times in a row. As in the pre‐switch trials, the colored shape was labeled for each trial (e.g., “Here is a blue one”), but no feedback was provided. Upon completing the post‐switch trials, children were shown a screen that had a smiley face with a thumbs up, and caregivers were asked to respond “Y” or “N” to a question asking if they pressed the keys for their child. The first matching dimension was counterbalanced across participants, such that participants either matched by shape then color, or by color then shape.

***Data integrity checks***. Upon completing the post‐switch trials, caregivers were asked to respond yes or no to the following questions: “Did you press the keys for your child?” and “Did you correct your child after the game switched to color (or shape)?” On the pre‐switch trials, there were very few non‐responses (ranging from 0 to 2 non‐responses, with 0 or 1 for most trials). On the post‐switch trials, there were more non‐responses, but the overall rate remained low (i.e., fewer than three non‐responses on most trials). Notably, on the first post‐switch trial there were eight (out of 93) non‐responses, consistent with an increase in conflict immediately following the switch, further suggesting the validity of this measure. Twenty‐two parents entered responses on behalf of their children as the study instructions allowed, and accuracy on the DCCS did not differ between these children and those who responded themselves, *p *> 0.45. Nine parents reported providing corrective feedback to their children in the post‐switch phase. We did not exclude these children from all analyses for two reasons. We aimed to avoid throwing out any valid data that these participants may have contributed (e.g., child activities data and temperament, first response in the post‐switch phase before receiving feedback) in order to conserve power to test our hypotheses and explore our data. We also aimed to avoid deviating from our preregistration and stated exclusion criteria. We thus analyze the relevant data with and without these participants included.

### 2.4 Analytic approach

We implemented our confirmatory analyses using generalized mixed‐effects regression via the *lme4* package (Bates et al., 2015) in R (R Core Team, 2014). We used glmer() to test predictors of whether a child performed accurately on a given post‐switch trial on the DCCS. Perseveration on the DCCS was operationalized as a continuous rather than categorical variable, with proportion of correct responses in the post‐switch phase taken to indicate the extent to which children were able to marshal executive control in order to avoid perseverative errors. Many preschool executive function tasks use this index (e.g., Day‐Night task, Grass‐Snow, Hand Game, Backward Digit Span; Carlson, 2005). The DCCS has tended to produce bimodal data, with many children sorting most cards correctly or incorrectly, hence, for pragmatic reasons, the dependent variable has tended to be modeled as categorical (e.g., “passing” or “failing,” according to some criterion). However, perseveration and flexibility have been theorized to be graded phenomena (Morton & Munakata, 2002; Perone et al., 2021). In this light, categorizing children as passing or failing (i.e., perseverating or not) risks throwing out meaningful variability, resulting in a dependent variable that is less sensitive. We address this by using logistic regression (predicting accuracy on a given post‐switch trial) in the context of linear mixed models, which can handle non‐normal distribution of responses.

Regression coefficients from the models are unstandardized and on the logit scale. Predictors in each of the models are our independent variable of interest (e.g., less‐structured time) and prespecified covariates (verbal skills, age, caregiver education (highest level completed), and family income). Caregiver education was modeled in two variables representing orthogonal contrasts (Masters vs. Ph.D. degree completed; any graduate degree vs. no graduate degree completed). Subject was modeled as a random factor, addressing repeated measurement of post‐switch performance (12 trials). Our analytic script and data file can be found here: https://osf.io/h4r6p/. As per our preregistration, these analyses only included children who accurately completed eight (75%) pre‐switch trials, which was necessary to establish a prepotent response.

## 3 RESULTS

### 3.1 How did children spend their time at home?

Caregivers’ estimates of the total amount of time children typically spent in various activities varied widely (*M* = 17.0 hours, *SD* = 5.9; excluding time spent sleeping and napping), consistent with the possibility that children were engaging in a large variety of activities that they did not engage in every single day. Thus, in all cases, estimates are interpreted as the time children regularly spent in a given activity, not time children spent in an activity every day.

**FIGURE 2:**
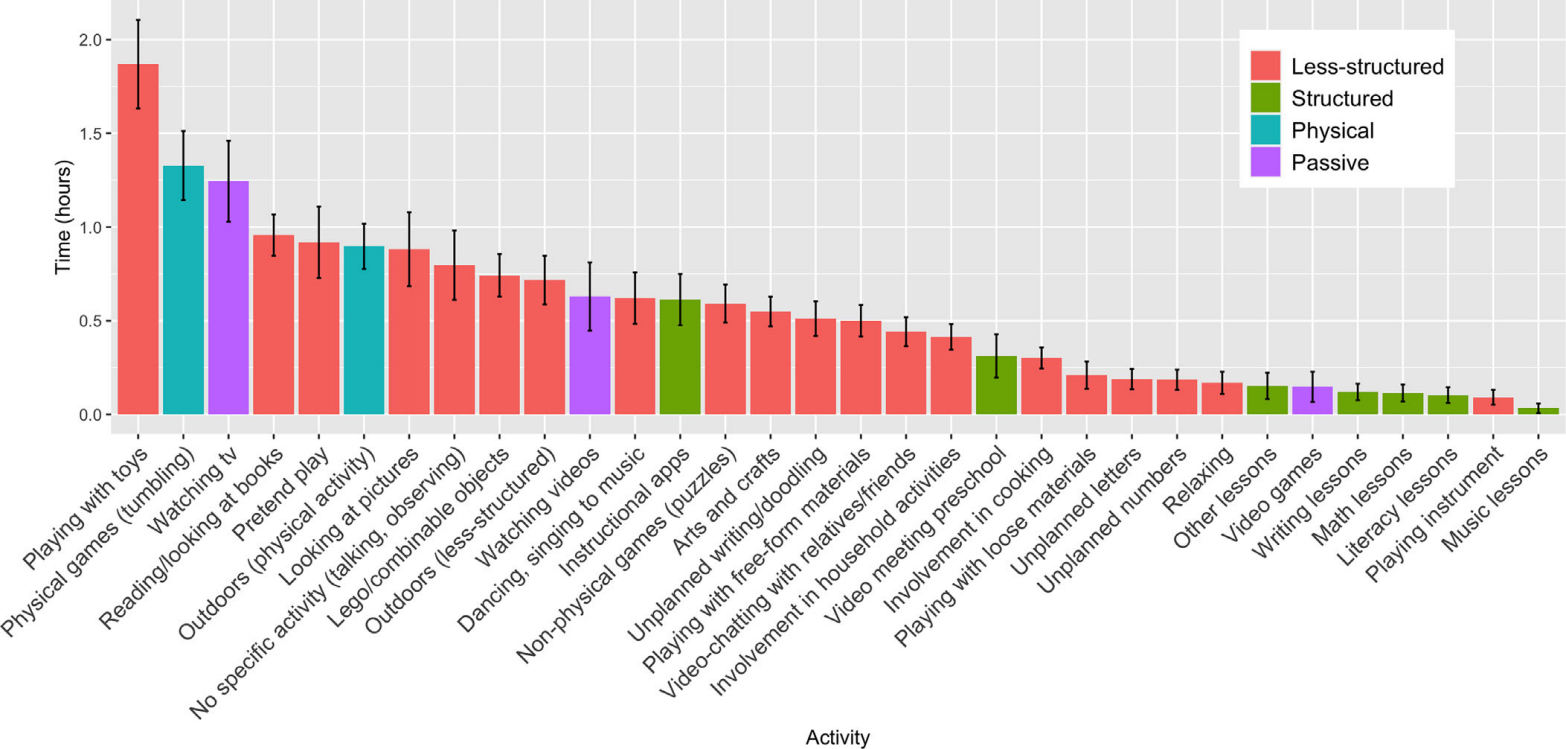
Children's time use as reported by their caregiver *Note*. Error bars represent 95% confidence intervals. Time in hours (*Y*‐axis) indicates the amount of time children tended to spend in an activity on a typical day.

Children engaged in a variety of activities while at home (Figure 2). Caregivers reported that the most time on a typical day was spent playing with toys (*M* = 1.9 hours, *SD* = 1.2). Children also spent a lot of time in physical play (*M* = 1.3 hours, *SD* = 0.9), watching television (*M* = 1.2 hours, *SD* = 1.1), reading or looking at books (*M* = 1.0 hours, *SD* = 0.5), pretending (*M* = 0.9 hours, *SD* = 0.9), going outside for a specific activity (e.g., riding a bike; *M* = 0.9 hours, *SD* = 0.6), and looking at pictures (*M* = 0.9 hours, *SD* = 1.0). At the other end of the scale, relatively little time was spent on lessons or less‐structured activities involving musical instruments (*M* = 0.1 hours, *SD* = 0.3), or structured lessons involving writing (*M* = 0.1 hours, *SD* = 0.2), reading (*M* = 0.1 hours, *SD* = 0.2), or numbers (*M* = 0.1 hours, *SD* = 0.2). As shown in Figure 2, children tended to spend time in a variety of less‐structured activities and physical activities and to spend more time in those activities versus other activities like lessons. This was true across a wide variety of activities.

### 3.2 How did caregivers’ engagement vary by activity?

**FIGURE 3:**
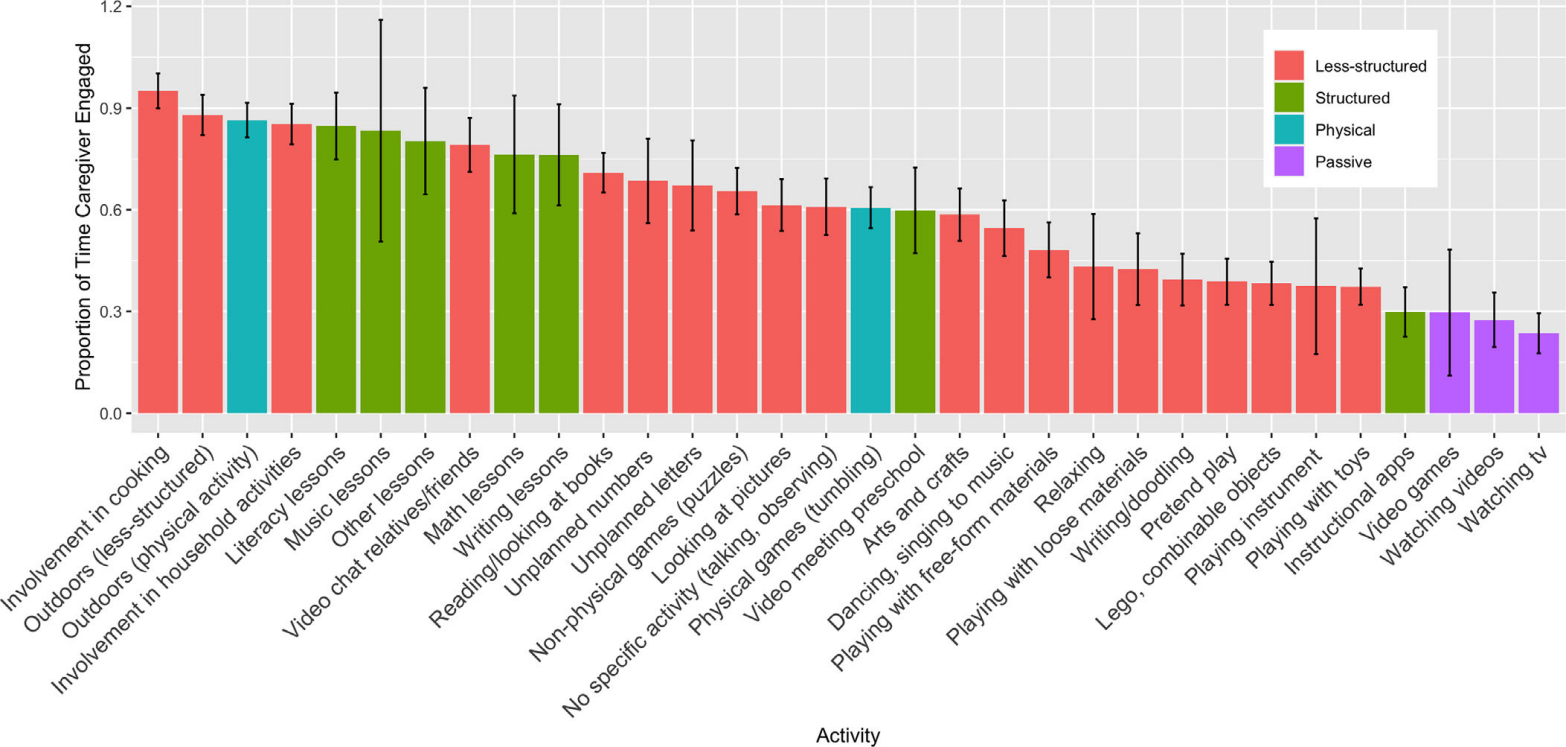
Proportion of time that caregiver was engaged in child's activity *Note*. Error bars represent 95% confidence intervals.

The amount of time caregivers spent engaged with their children in activities also varied by activity (Figure 3). As one might expect, caregivers were most engaged in activities that required supervision, including cooking (*M* = 95%, *SD* = 19%), playing outside (*M* = 87%, *SD* = 25%), household activities (*M* = 85%, *SD* = 24%), or structured lessons (*M* = 80%, *SD* = 34%). They were least involved in passive activities like TV watching (*M* = 24%, *SD* = 25%) and watching videos (*M* = 28%, *SD* = 29%). They were also not very involved in children's play with toys (*M* = 37%, *SD* = 25%). They tended to be involved in activities like reading books (*M* = 71%, *SD* = 28%), playing non‐physical games (*M* = 65%, *SD* = 29%), looking at pictures (*M* = 61%, *SD* = 32%), and unplanned activities with letters (*M* = 67%, *SD* = 38%). Exploratory analyses indicated that caregivers spent proportionally more time engaged with their children during less‐structured versus structured activities, *b* = 0.08, *SE* = 0.04, *t*(77) = 2.12, *p* = 0.037.

### 3.3 How did children's time use relate to performance on the DCCS?

Performance on the DCCS was consistent with what has been found in the laboratory, with children showing a performance decrement after the switch (pre‐switch *M* = 0.97, *SD* = 0.06; post‐switch *M* = 0.87, *SD* = 0.22). Moreover, children showed significant age‐related improvement on the task, *r* = 0.27, *p* = 0.001, replicating a robust finding across labs (Doebel & Zelazo, 2015). Of the children who were accurate on 75% or more of the pre‐switch trials (89 of 93), 39 were accurate on all 12 post‐switch trials and only one was inaccurate on all post‐switch trials. Fifty‐two were accurate on more than one but less than 12 trials. Our results are consistent with prior research showing variability across and within studies in how well younger children perform, with some samples of 3‐year‐olds sorting most cards correctly in the post‐switch phase of the standard DCCS (Doebel & Zelazo, 2015).

As hypothesized, caregivers’ estimates of the typical amount of time their child spent in less‐structured activities were associated with post‐switch trial accuracy on the DCCS, *b* = 0.13, *SE* = 0.06, *z* = 2.11, *p* = 0.035 (Table 1), over and above age, family income, caregiver education, and verbal skills. These results held when we excluded children (*n* = 9) whose caregivers reported correcting their child's responses in the post‐switch phase (*p* = 0.022) and also held when the planned covariates were not included (with all children: *p* = 0.053; without nine children whose parents corrected them: *p* = 0.033).

**TABLE 1:** Summary of linear mixed models predicting post‐switch trial accuracy on the DCCS

Model	*b*	*SE*	*z*	*p*	*OR*	*Lower CI*	*Upper CI*
Less‐structured activities							
Intercept	−1.79	1.95	−0.92	0.359	0.17	0.00	7.64
Less‐structured time	0.13	0.06	2.11	0.035*	1.14	1.01	1.29
Child age	0.11	0.04	3.26	0.001**	1.12	1.05	1.20
Ph.D. vs. Master's degree	0.27	0.77	0.35	0.729	1.31	0.29	5.90
Grad degree vs. no grad degree	0.14	0.65	0.21	0.833	1.15	0.32	4.14
Family income	−0.15	0.16	−0.98	0.328	0.86	0.63	1.17
DVAP	−0.01	0.01	−1.30	0.193	0.99	0.97	1.01
Structured activities							
Intercept	−0.05	1.79	−0.03	0.976	0.95	0.03	31.56
Structured time	−0.09	0.24	−0.39	0.694	0.91	0.57	1.45
Child age	0.12	0.04	3.04	0.002**	1.13	1.04	1.22
Ph.D. vs. Master's degree	0.27	0.77	0.35	0.724	1.31	0.29	5.98
Grad degree vs. no grad degree	−0.06	0.65	−0.09	0.927	0.94	0.26	3.38
Family income	−0.20	0.16	−1.23	0.219	0.82	0.60	1.12
DVAP	−0.01	0.01	−1.40	0.161	0.99	0.97	1.01
Passive activities							
Intercept	0.80	1.75	0.46	0.648	2.22	0.07	68.07
Passive time	−0.33	0.21	−1.62	0.105	0.72	0.48	1.07
Child age	0.12	0.04	3.33	0.001**	1.13	1.05	1.21
Ph.D. vs. Master's degree	0.34	0.77	0.44	0.659	1.40	0.31	6.30
Grad degree vs. no grad degree	−0.23	0.66	−0.35	0.724	0.79	0.22	2.87
Family income	−0.18	0.16	−1.15	0.249	0.83	0.61	1.14
DVAP	−0.02	0.01	−1.59	0.112	0.98	0.96	1.00

*Note. b* = unstandardized coefficients; *DVAP* = Developmental Vocabulary Assessment for Parents; *SE* = standard error; *OR* = odds ratio; *CI* = 95% confidence interval. *p < 0.05, **p < 0.01, ***p < 0.001.

**FIGURE 4:**
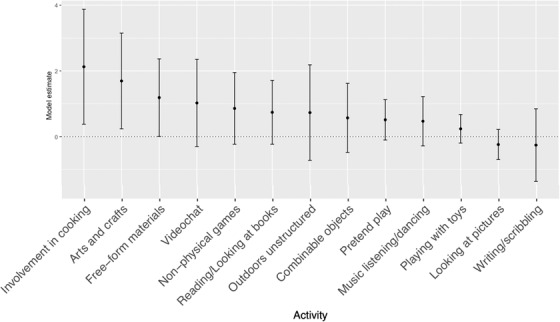
Relations between specific less‐structured activities and rate of successful switching on the DCCS *Note*. Each plotted estimate is from a separate generalized linear mixed model with age, family income, caregiver education, and verbal skills included as covariates (like confirmatory models). Estimates are unstandardized coefficients. Error bars represent 95% confidence intervals.

We then implemented a second, exploratory analysis aimed at assessing whether the total number of less‐structured activities that children engaged in was associated with performance on the DCCS. Children varied in how many different less‐structured activities they typically spent any amount of time in (range: 4–19 activities; *M* = 12.78, *SD* = 3.43), and we found that the larger the number of activities, the more successful children were at switching on the DCCS, *b* = 0.17, *SE* = 0.08, *z* = 2.01, *p* = 0.045, controlling for the same covariates as in our confirmatory model (*p* = 0.025 when excluding children whose caregivers corrected their post‐switch performance). To gain preliminary insight into how less‐structured time might support executive function, we report which less‐structured activities tended to be more closely related to performance on the DCCS (Figure 4).

We also hypothesized that more time spent using passive visual media (TV, YouTube, video games) would be negatively associated with executive function task performance. Our confirmatory test was not significant (*b* = −0.33, *SE* = 0.21, *z* = −1.62, *p* = 0.105; Table 1); however, excluding the nine children whose caregivers corrected their performance resulted in a trend that was consistent with our hypothesis, *b* = −0.43, *SE* = 0.23, *z* = −1.83, *p* = 0.067.

Our results did not support the hypothesis that more time spent in structured lessons would be positively associated with executive function task performance, *b* = −0.09, *SE* = 0.24, *z* = −0.39, *p* = 0.694 (Table 1). We also did not find evidence that time spent in primarily physical activity was related to performance on the DCCS, *b* = 0.03, SE = 0.21, *z* = 0.13, *p* = 0.894 (without nine participants whose caregivers corrected them during post‐switch phase: *b* = 0.42, *SE* = 0.30, *z* = 1.4, *p* = 0.16).

### 3.4 How did caregivers’ perceptions of their child's temperament relate to how their child's time was organized?

Caregivers’ perceptions of their child's temperament were associated with how children spent their time. We ran four multiple regression analyses for each time use category and included the following predictors: temperament variables (effortful control, surgency, and negative affect), sex, family income, and caregiver education (Table 2). Time in less‐structured activities and primarily physical activities was positively associated with effortful control (*p* = 0.007 and *p* = 0.010, respectively). Time engaged with passive media, by contrast, was positively associated with negative affect (*p* = 0.003), surgency (*p* = 0.017), sex (more for boys; *p* = 0.021), and caregiver education (more for children of graduate‐educated parents; *p* = 0.049). Time in structured activities was not associated with any temperament variables.

**TABLE 2:** Summary of four exploratory linear models of predictors of children's time use

Model	*B*	*SE*	*t*	*p*	*L. CI*	*U. CI*
Less‐structured activities						
Intercept	0.02	6.17	0.00	0.998	−12.28	12.31
Child age	−0.04	0.06	−0.59	0.558	−0.16	0.09
Sex	−0.58	0.53	−1.10	0.276	−1.62	0.47
Effortful control	2.11	0.76	2.77	0.007**	0.59	3.63
Negative affect	0.64	0.64	0.99	0.327	−0.65	1.92
Surgency	0.54	0.62	0.87	0.386	−0.70	1.79
DVAP	−0.00	0.02	−0.14	0.892	−0.04	0.04
Income	−0.31	0.30	−1.06	0.294	−0.90	0.28
Ph.D. vs. Master's degree	0.36	1.50	0.24	0.810	−2.63	3.36
Grad degree vs. no grad degree	−0.68	1.18	−0.58	0.563	−3.02	1.66
Structured activities						
Intercept	−3.77	1.58	−2.39	0.020*	−6.91	−0.62
Child age	0.07	0.02	4.17	<0.001***	0.03	0.10
Sex	−0.06	0.13	−0.44	0.663	−0.33	0.21
Effortful control	0.22	0.19	1.11	0.273	−0.17	0.60
Negative affect	0.13	0.16	0.80	0.427	−0.20	0.46
Surgency	0.03	0.16	0.17	0.866	−0.29	0.34
DVAP	−0.00	0.01	−0.86	0.392	−0.01	0.01
Income	0.06	0.08	0.79	0.431	−0.09	0.21
Ph.D. vs. Master's degree	−0.11	0.38	−0.29	0.775	−0.88	0.66
Grad degree vs. no grad degree	0.11	0.30	0.36	0.718	−0.49	0.71
Passive activities						
Intercept	0.23	1.67	0.14	0.890	−3.10	3.56
Child age	−0.01	0.02	−0.39	0.700	−0.04	0.03
Sex	−0.33	0.14	−2.35	0.021*	−0.62	−0.05
Effortful control	−0.30	0.21	−1.46	0.149	−0.71	0.11
Negative affect	0.53	0.17	3.05	0.003**	0.18	0.88
Surgency	0.41	0.17	2.44	0.017*	0.08	0.75
DVAP	−0.01	0.01	−1.29	0.203	−0.02	0.00
Income	0.09	0.08	1.07	0.289	−0.07	0.24
Ph.D. vs. Master's degree	0.52	0.41	1.27	0.209	−0.30	1.33
Grad degree vs. no grad degree	−0.64	0.32	−2.00	0.049*	−1.27	−0.00
Physical activities						
Intercept	−2.07	1.75	−1.19	0.239	−5.55	1.41
Child age	0.01	0.02	0.69	0.490	−0.02	0.05
Sex	−0.03	0.15	−0.17	0.863	−0.32	0.27
Effortful control	0.57	0.22	2.66	0.010*	0.14	1.00
Negative affect	0.00	0.18	0.02	0.983	−0.36	0.37
Surgency	0.26	0.18	1.50	0.139	−0.09	0.62
DVAP	0.00	0.01	0.02	0.988	−0.01	0.01
Income	−0.08	0.08	−0.90	0.369	−0.24	0.09
Ph.D. vs. Master's degree	0.32	0.43	0.74	0.461	−0.53	1.16
Grad degree vs. no grad degree	0.33	0.33	0.99	0.323	−0.33	0.99

*Note. b* = unstandardized coefficients; *DVAP* = Developmental Vocabulary Assessment for Parents; *SE* = standard error; *OR* = odds ratio; *CI* = 95% confidence intervals.

Finally, children's activities varied by age and sex. Age was positively associated with time in structured activities (*b* = 0.07, *SE* = 0.02, *t*(89) = 4.17, *p* < 0.001), and this was true for most activities within this category (see Table S1 in the supplemental materials for the full activity model analyses). For less‐structured activities, unstructured writing increased with age (*b* = 0.01, *SE* = 0.00, *t*(89) = 3.30, *p* = 0.001). Overall, age was not a predictor of time spent in less‐structured activities, but more fine‐grained exploration indicated time playing with freeform materials (e.g., playdoh) decreased with age (*b* = −0.01, *SE* = 0.00, *t*(89) = −2.34, *p* = 0.022), and time playing video games increased with age (*b* = 0.01, *SE* = 0.00, *t*(88) = 2.49, *p* = 0.014). The only additional notable sex differences were that girls spent more time engaged in arts and crafts (*b* = −1.16, *SE* = 0.04, *t*(88) = −4.08, *p* < 0.001) and playing with freeform materials (*b* = −0.13, *SE* = 0.04, *t*(89) = −3.07, *p* = 0.003), and less time playing with combinable objects (e.g., Lego; *b* = 0.18, *SE* = 0.06, *t*(89) = 3.16, *p* = 0.002).

## 4 DISCUSSION

The reported study yields rich data providing new insights about the relation between children's time use and their cognitive skill development. Time and variety in less‐structured activities, excluding primarily physical activity and engagement with passive media, were associated with successful switching on a measure of cued executive function, the DCCS, controlling for age, family income, caregiver education, and a measure of verbal knowledge. We thus show that the relation between less‐structured time and executive function is not specific to self‐directed executive function, as previously suggested (Barker et al., 2014).

These findings are consistent with several possibilities. One is that time in less‐structured activities benefits executive function by providing opportunities to acquire valued knowledge that helps children engage executive function in culturally valued ways. On the other hand, it is also possible that less‐structured activities helped children strengthen executive function broadly, rather than providing knowledge that supports using it in particular ways (e.g., on the DCCS). The reported study is the first to demonstrate a relation between less‐structured time and externally cued executive function and was motivated by ideas regarding the role of knowledge in performance on tasks like the verbal fluency task and the DCCS (Doebel, 2020). Prior experimental work suggests that knowledge matters (e.g., Bardikoff & Sabbagh, 2021; Doebel & Zelazo, 2016; Perone et al., 2015); however, it is possible that less‐structured activities provide children with opportunities to practice and strengthen self‐directed and cued executive function in a general way, regardless of the specific knowledge required in a task. More research is needed to adjudicate between these competing accounts.

Conversely, it could be that children with better executive function are more likely to show interest in less‐structured activities, or that children with better executive function had parents with better executive function, who, in turn, were more likely to facilitate children's less‐structured activities. Future research can test competing hypotheses with experimental and longitudinal designs.

We also found that caregivers tended to spend more time with their children during less‐structured versus structured activities. Many less‐structured activities that tended to relate to DCCS performance (e.g., being involved in cooking activities, engaging in arts and crafts, completing puzzles and matching games, pretending) could represent proxies for a certain kind of quality of time that supports language and conceptual acquisition, learning about others’ minds and expectations, and learning about following rules and instructions, all of which may support engaging control on the DCCS and other executive function tasks (Alderson‐Day & Fernyhough, 2015; Bardikoff & Sabbagh, 2021; Bernier et al., 2010; Carlson & Moses, 2001; White & Carlson, 2021). Testing relations between specific kinds of activities, knowledge, and executive function skills is thus a key direction for future research.

We expected that structured time might be associated with executive function performance, but we did not find support for this hypothesis. This could be because such structured time indeed restricts the potential to acquire diverse knowledge that, in turn, benefits using executive function. On the other hand, the absence of support for our hypothesis may also be explained by the overall low incidence of structured activities in this sample.

We also explored how caregivers’ perceptions of their child's temperament related to how their child's time was spent. Children rated higher in effortful control, a general tendency towards emotion and behavioral regulation, were more likely to spend time engaged in physical or less‐structured activities. They also engaged in a greater variety of less‐structured activities. Children rated higher in negative affect or positive emotional reactivity (surgency) were more likely to spend time engaged with passive media. These findings suggest that how caregivers perceive their children may lead them to expand or limit children's time in certain activities, affecting opportunities to learn. On the other hand, children with certain dispositions may pursue and engage longer in specific kinds of activities. Future research can test these different possibilities. For example, if caregivers’ perceptions are important determinants of how children spend their time, intervention research could target those perceptions and their antecedents to shift children's time use in constructive directions.

Our findings related to passive media use and executive function were inconclusive in that time spent engaged with passive media did not significantly predict switching on the DCCS, yet there was a trend in the expected direction. It is possible, for example, that there was a small effect, and our study was underpowered to detect it. Future work can address the extent to which passive media use may restrict learning opportunities that could benefit children's developing executive function skills.

Additional explorations yielded noteworthy patterns related to age and sex. Whereas child age did not predict time spent in less‐structured, passive, or primarily physical activities overall, several activities within the less‐structured and passive categories tended to increase with age (playing videogames and unstructured writing/doodling), while others decreased (time using freeform materials like Play Doh). Further, while sex did not predict time spent in less‐structured, passive, or physical activities overall, girls tended to spend more time in some activities, including arts and crafts and using freeform materials, as compared to boys, who tended to spend more time using combinable objects. Future research can confirm such patterns and explore implications for developing executive function.

Our study also suggests that it is feasible to conduct remote studies involving behavioral data collection with preschoolers online. However, such research is not without challenges. Recruitment can be difficult for an online study with a duration above 30 minutes. Families may be less willing to participate in the same studies in which they would gladly participate in person, possibly because in‐lab participation offers distinct rewards (e.g., opportunities to observe child engaging with an experimenter, opportunities to chat with researchers).

We note some limitations of the reported study. It is important to note that families in our sample were, on average, highly educated and affluent. These findings provide a starting point for future research on this topic that can explore these questions in a more diverse sample of families, as there is a pressing need to better understand how diverse children are spending their time at home and how this relates to their emerging executive function skills, which are known to be a key ingredient in school‐readiness (Blair & Razza, 2007).

It is also important to consider that our study was conducted during a time of global crisis that must be considered when attempting to generalize to different times and circumstances. It is possible that the patterns documented here do not reflect these children's typical experiences at home prior to COVID‐19.

The methods used in the reported study do not allow us to speak to whether the degree of structure a child is provided with in various activities is related to externally cued executive function. This is because we measured the variety and amount of time children spent in a broad range of activities classified as less‐structured and did not code the degree of structure that characterized each activity. Such coding presents a host of challenges that may be overcome in future research. For example, while we construed children's involvement in housework as a less‐structured activity (Barker et al., 2014), one could argue that this activity could involve more or less imposed structure, depending on the caregiver. Future research could address this by asking caregivers about the amount of structure and choice provided in specific activities.

The reported study advances theory on executive function by articulating and beginning to test new ideas about how experience is related to emerging executive function skills. Research on the role of experience in the development of executive function has often been guided by a model of executive function as a small set of general neurocognitive abilities or processes. This kind of model constrains how one thinks about the role of experience (e.g., as influencing healthy brain development that supports executive processes). The reported study begins to explore a different way of thinking about how experience may be related to developing executive function skills (i.e., by providing knowledge and skills that support using executive function in particular ways).

How young children spend their time matters for development and school readiness, and thus is an important area of study, particularly at a time when many children are spending more time at home. This research shows that time and variety in less‐structured activities is not only associated with self‐directed executive function, as previously found, but also externally cued executive function in younger children. Caregivers’ ratings of children's temperament are also associated with how children spend their time. Future research can further explore these patterns with more diverse samples and causal designs to better understand what kinds of less‐structured activities may be particularly valuable for preparing children to thrive in the world.

## CONFLICT OF INTEREST

The authors have no conflicts of interest to report.

## AUTHOR CONTRIBUTIONS

NJS and SD developed the study concept and design, conducted all analyses, and drafted the manuscript. GS developed the online tasks and provided feedback on the study concept and manuscript draft. All authors approved the final submission.

## Supporting information

Supporting Information

## Data Availability

The data that support the findings of this study are available via the Open Science Framework here: https://osf.io/3k49g/

## References

[desc13226-bib-0001] Alderson‐Day, B. , & Fernyhough, C. (2015). Inner speech: Development, cognitive functions, phenomenology, and neurobiology. Psychological Bulletin, 141(5), 931‐965. 10.1037/bul0000021 26011789 PMC4538954

[desc13226-bib-0002] Bardikoff, N. , & Sabbagh, M. A. (2021). Multidimensional reasoning can promote 3‐year‐old children's performance on the Dimensional Change Card Sort Task. Child Development. 10.1111/cdev.13533 33496007

[desc13226-bib-0003] Barker, J. E. , Semenov, A. D. , Michaelson, L. , Provan, L. S. , Snyder, H. R. , & Munakata, Y. (2014). Less‐structured time in children's daily lives predicts self‐directed executive functioning. Frontiers in Psychology, 5, 593. 10.3389/fpsyg.2014.00593 25071617 PMC4060299

[desc13226-bib-0004] Bates, D. , Mächler, M. , Bolker, B. , & Walker, S. (2015). Fitting linear mixed‐effects models using lme4. Journal of Statistical Software, 67(1), 1‐48. 10.18637/jss.v067.i01

[desc13226-bib-0005] Becker, D. R. , McClelland, M. M. , Loprinzi, P. , & Trost, S. G. (2014). Physical activity, self‐regulation, and early academic achievement in preschool children. Early Education & Development, 25(1), 56‐70.

[desc13226-bib-0006] Bernier, A. , Carlson, S. M. , & Whipple, N. (2010). From external regulation to self‐regulation: Early parenting precursors of young children's executive functioning. Child Development, 81(1), 326‐339. 10.1111/j.1467-8624.2009.01397.x 20331670

[desc13226-bib-0007] Blair, C. , & Razza, R. P. (2007). Relating effortful control, executive function, and false belief understanding to emerging math and literacy ability in kindergarten. Child Development, 78(2), 647‐663. 10.1111/j.1467-8624.2007.01019.x 17381795

[desc13226-bib-0008] Buss, A. T. , & Spencer, J. P. (2014). The emergent executive: A dynamic field theory of the development of executive function. Monographs of the Society for Research in Child Development, 79(2), vii–103. 24818836 10.1002/mono.12096 PMC4426851

[desc13226-bib-0009] Carlson, S. M. (2005). Developmentally sensitive measures of executive function in preschool children. Developmental Neuropsychology, 28(2), 595‐616. 10.1207/s15326942dn2802_3 16144429

[desc13226-bib-0010] Carlson, S. M. , & Moses, L. J. (2001). Individual differences in inhibitory control and children's theory of mind. Child Development, 72(4), 1032‐1053. 10.1111/1467-8624.00333 11480933

[desc13226-bib-0011] Carlson, S. M. , & Zelazo, P. D. (2014). Minnesota executive function scale: Test manual. Reflection Sciences, Inc. St. Paul, MN.

[desc13226-bib-0013] Cook, C. J. , Howard, S. J. , Scerif, G. , Twine, R. , Kahn, K. , Norris, S. A. , & Draper, C. E. (2019). Associations of physical activity and gross motor skills with executive function in preschool children from low‐income South African settings. Developmental Science, 22(5), 1‐13. 10.1111/desc.12820 30801916

[desc13226-bib-0014] Doebel, S. (2020). Rethinking executive function and its development. Perspectives on Psychological Science, 15(4), 942‐956. 10.1177/1745691620904771 32348707

[desc13226-bib-0015] Doebel, S. , & Zelazo, P. D. (2013). Bottom‐up and top‐down dynamics in young children's executive function: Labels aid 3‐year‐olds’ performance on the Dimensional Change Card Sort. Cognitive development, 28(3), 222‐232. 10.1016/j.cogdev.2012.12.001 24882942 PMC4039630

[desc13226-bib-0016] Doebel, S. , & Zelazo, P. D. (2015). A meta‐analysis of the Dimensional Change Card Sort: Implications for developmental theories and the measurement of executive function in children. Developmental Review, 38, 241‐268. 10.1016/j.dr.2015.09.001 26955206 PMC4778090

[desc13226-bib-0017] Doebel, S. , & Zelazo, P. D. (2016). Seeing conflict and engaging control: Experience with contrastive language benefits executive function in preschoolers. Cognition, 157, 219‐226. 10.1016/j.cognition.2016.09.010 27658118 PMC5143180

[desc13226-bib-0018] Fletcher, A. C. , Nickerson, P. , & Wright, K. L. (2003). Structured leisure activities in middle childhood: Links to well‐being. Journal of Community Psychology, 31(6), 641‐659. 10.1002/jcop.10075

[desc13226-bib-0019] Gray, P. (2013). Free to learn: Why unleashing the instinct to play will make our children happier, more self‐reliant, and better students for life. Basic Books.

[desc13226-bib-0020] Hirsh‐Pasek, K. , Golinkoff, R. M. , Berk, L. E. , & Singer, D. (2009). A mandate for playful learning in preschool: Applying the scientific evidence. Oxford University Press.

[desc13226-bib-0056] Korucu, I. , Rolan, E. , Napoli, A. R. , Purpura, D. J. , & Schmitt, S. A. (2019). Development of the Home Executive Function Environment (HEFE) Scale: Assessing its relation to preschoolers' executive function. Early Childhood Research Quarterly, 47, 9‐19. 10.1016/j.ecresq.2018.09.001

[desc13226-bib-0021] Korucu, I. , & Schmitt, S. A. (2020). Continuity and change in the home environment: Associations with school readiness. Early Childhood Research Quarterly, 53, 97‐107. 10.1016/j.ecresq.2020.03.002

[desc13226-bib-0022] Lakens, D. (2016). Why you don't need to adjust your alpha level for all tests you'll do in your lifetime. Blog post, http://daniellakens.blogspot.com/2016/02/why‐you‐dont‐need‐to‐adjust‐you‐alpha.html

[desc13226-bib-0023] Larson, R. W. , & Verma, S. (1999). How children and adolescents spend time across the world: Work, play, and developmental opportunities. Psychological Bulletin, 125(6), 701. 10.1037/0033-2909.125.6.701 10589300

[desc13226-bib-0024] Libertus, M. E. , Odic, D. , Feigenson, L. , & Halberda, J. (2015). A developmental vocabulary assessment for parents (DVAP): Validating parental report of vocabulary size in 2‐ to 7‐year‐old children. Journal of Cognition and Development, 16(3), 442‐454. 10.1080/15248372.2013.835312

[desc13226-bib-0025] Lillard, A. S. (2017). Montessori: The science behind the genius. Oxford University Press.

[desc13226-bib-0026] Lillard, A. S. , Drell, M. B. , Richey, E. M. , Boguszewski, K. , & Smith, E. D. (2015). Further examination of the immediate impact of television on children's executive function. Developmental Psychology, 51(6), 792‐805. 10.1037/a0039097 25822897

[desc13226-bib-0027] Lillard, A. S. , Heise, M. J. , Richey, E. M. , Tong, X. , Hart, A. , & Bray, P. M. (2017). Montessori preschool elevates and equalizes child outcomes: A longitudinal study. Frontiers in Psychology, 8, 1‐19. 10.3389/fpsyg.2017.01783 29163248 PMC5670361

[desc13226-bib-0028] Lillard, A. , & Else‐Quest, N. (2006). The early years: Evaluating Montessori education. Science, 313(5795), 1893‐1894. 10.1126/science.1132362 17008512

[desc13226-bib-0030] Meeks, C. B. , & Mauldin, T. (1990). Children's time in structured and unstructured leisure activities. Lifestyles Family and Economic Issues, 11(3), 257‐281. 10.1007/bf00987003

[desc13226-bib-0031] Melhuish, E. C. , Sylva, K. , Sammons, P. , Siraj‐Blatchford, I. , Taggart, B. , Phan, M. , & Malin, A. (2008). Preschool influences on mathematics achievement. Science, 321(5893), 1161‐1162. 10.1126/science.1158808 18755959

[desc13226-bib-0032] Morton, J. B. , & Munakata, Y. (2002). Active versus latent representations: A neural network model of perseveration, dissociation, and decalage. Developmental Psychobiology: The Journal of the International Society for Developmental Psychobiology, 40(3), 255‐265. 10.1002/dev.10033 11891637

[desc13226-bib-0033] Ng, F. F. , & Wei, J. (2020). Delving into the minds of Chinese parents: What beliefs motivate their learning‐related practices? Child Development Perspectives, 14(1), 61‐67. 10.1111/cdep.12358

[desc13226-bib-0034] Nieto‐López, M. , Sanchez‐Lopez, M. , Visier‐Alfonso, M. E. , Martinez‐Vizcaino, V. , Jimenez‐Lopez, E. , & Alvarez‐Bueno, C. (2020). Relation between physical fitness and executive function variables in a preschool sample. Pediatric Research, 88(4), 623‐628. 10.1038/s41390-020-0791-z 32000261

[desc13226-bib-0036] Perone, S. , Molitor, S. J. , Buss, A. T. , Spencer, J. P. , & Samuelson, L. K. (2015). Enhancing the executive functions of 3‐year‐olds in the dimensional change card sort task. Child Development, 86(3), 812‐827. 10.1111/cdev.12330 25441395 PMC4646608

[desc13226-bib-0037] Perone, S. , Simmering, V. R. , & Buss, A. T. (2021). A dynamic reconceptualization of executive‐function development. Perspectives on Psychological Science, 1‐33. 10.1177/1745691620966792 PMC8364921 33593126

[desc13226-bib-0038] Putnam, S. P. , & Rothbart, M. K. (2006). Development of short and very short forms of the Children's Behavior Questionnaire. Journal of Personality Assessment, 87(1), 102‐112. 10.1207/s15327752jpa8701_09 16856791

[desc13226-bib-0039] R. Core Team . (2014). R: A language and environment for statistical computing. R Foundation for Statistical Computing.

[desc13226-bib-0040] Rege, M. , Størksen, I. , Solli, I. F. , Kalil, A. , McClelland, M. , Braak, D. T. , Lenes, R. , Lunde, S. , Breive, S. , Carlsen, M. , Erfjord, I. , & Hundeland, P. S. (2019). *Promoting child development in a universal preschool system: A field experiment* (CESifo Working Paper no. 7775). Munich Society for the Promotion of Economic Research. https://www.cesifo.org/node/44736

[desc13226-bib-0042] Rodriguez, E. T. , & Tamis‐LeMonda, C. S. (2011). Trajectories of the home learning environment across the first 5 years: Associations with children's vocabulary and literacy skills at prekindergarten. Child Development, 82(4), 1058‐1075. 10.1111/j.1467-8624.2011.01614.x 21679179

[desc13226-bib-0043] Rosen, M. L. , Hagen, M. P. , Lurie, L. A. , Miles, Z. E. , Sheridan, M. A. , Meltzoff, A. N. , & McLaughlin, K. A. (2020). Cognitive stimulation as a mechanism linking socioeconomic status with executive function: A longitudinal investigation. Child Development, 91(4), 762‐779. 10.1111/cdev.13315 PMC7138720 31591711

[desc13226-bib-0045] Rubin, M. (2021). When to adjust alpha during multiple testing: a consideration of disjunction, conjunction, and individual testing. Synthese, 199(3‐4), 10969‐11000. 10.1007/s11229-021-03276-4

[desc13226-bib-0046] Son, S. H. , & Morrison, F. J. (2010). The nature and impact of changes in home learning environment on development of language and academic skills in preschool children. Developmental Psychology, 46(5), 1103. 10.1037/a0020065 20822226

[desc13226-bib-0047] Stoet, G. (2010). PsyToolkit: A software package for programming psychological experiments using Linux. Behavior research methods, 42(4), 1096‐1104. 10.3758/BRM.42.4.1096 21139177

[desc13226-bib-0048] Stoet, G. (2017). PsyToolkit: A novel web‐based method for running online questionnaires and reaction‐time experiments. Teaching of Psychology, 44(1), 24‐31. 10.1177/0098628316677643

[desc13226-bib-0050] White, R. E. , & Carlson, S. M. (2021). Pretending with realistic and fantastical stories facilitates executive function in 3‐year‐old children. Journal of Experimental Child Psychology, 207, 1‐17. 10.1016/j.jecp.2021.105090 PMC8146118 33684892

[desc13226-bib-0051] Wiebe, S. A. , Sheffield, T. D. , & Espy, K. A. (2012). Separating the fish from the sharks: A longitudinal study of preschool response inhibition. Child Development, 83(4), 1245‐1261. 10.1111/j.1467-8624.2012.01765.x 22533453 PMC3399978

[desc13226-bib-0052] Zelazo, P. D. (2006). The Dimensional Change Card Sort (DCCS): A method of assessing executive function in children. Nature Protocols, 1(1), 297‐301. 10.1038/nprot.2006.46 17406248

[desc13226-bib-0053] Zelazo, P. D. (2015). Executive function: Reflection, iterative reprocessing, complexity, and the developing brain. Developmental Review, 38, 55‐68. 10.1016/j.dr.2015.07.001

[desc13226-bib-0054] Zelazo, P. D. , Anderson, J. E. , Richler, J. , Wallner‐Allen, K. , Beaumont, J. L. , & Weintraub, S. (2013). II. NIH Toolbox Cognition Battery (CB): Measuring executive function and attention. Monographs of the Society for Research in Child Development, 78(4), 16‐33. 10.1111/mono.12032 23952200

