# Supplementary material for: What are the kids doing? Exploring young children's activities at home and relations with externally cued executive function and child temperament
Source: Dev Sci. 2022 Jan 23;25(5):e13226. doi: 10.1111/desc.13226 (PMC9540249; doi:10.1111/desc.13226)
Supplement: Supplementary file 1 — Supporting Information [file DESC-25-e13226-s001.docx]

**Supplementary Materials**

**S1 Table. Exploratory Models of Children’s Activities and Age, Sex.**

|  |  |  |  |  | *95% CI* | |
| --- | --- | --- | --- | --- | --- | --- |
| Model | *b* | *SE* | *t* | *p* | *LL* | *UL* |
| Arts and crafts |  |  |  |  |  |  |
| Intercept | 0.39 | 0.20 | 2.01 | 0.048 | 0.00 | 0.78 |
| Child age | 0.00 | 0.00 | 0.87 | 0.387 | -0.00 | 0.01 |
| Sex | -0.16 | 0.04 | -4.08 | **<0.001** | -0.23 | -0.08 |
| Dancing and singing to music |  |  |  |  |  |  |
| Intercept | 0.83 | 0.37 | 2.25 | **0.027** | 0.10 | 1.57 |
| Child age | -0.00 | 0.01 | -0.57 | 0.568 | -0.02 | 0.01 |
| Sex | -0.09 | 0.07 | -1.29 | 0.200 | -0.24 | 0.05 |
| Helping with cooking |  |  |  |  |  |  |
| Intercept | 0.53 | 0.15 | 3.59 | **0.001** | 0.24 | 0.83 |
| Child age | -0.00 | 0.00 | -1.58 | 0.118 | -0.01 | 0.00 |
| Sex | -0.06 | 0.03 | -1.94 | 0.056 | -0.11 | 0.00 |
| Household activities |  |  |  |  |  |  |
| Intercept | 0.21 | 0.18 | 1.13 | 0.263 | -0.16 | 0.57 |
| Child age | 0.00 | 0.00 | 1.15 | 0.253 | -0.00 | 0.01 |
| Sex | -0.02 | 0.04 | -0.56 | 0.577 | -0.09 | 0.05 |
| Instructional apps |  |  |  |  |  |  |
| Intercept | 0.06 | 0.37 | 0.17 | 0.866 | -0.67 | 0.79 |
| Child age | 0.01 | 0.01 | 1.55 | 0.125 | 0.00 | 0.02 |
| Sex | -0.01 | 0.07 | -0.19 | 0.851 | -0.16 | 0.13 |
| Lessons – Literacy |  |  |  |  |  |  |
| Intercept | -0.11 | 0.11 | -0.96 | 0.342 | -0.33 | 0.11 |
| Child age | 0.00 | 0.00 | 1.92 | 0.058 | 0.00 | 0.01 |
| Sex | 0.02 | 0.02 | 0.74 | 0.463 | -0.03 | 0.06 |
| Lessons – Math |  |  |  |  |  |  |
| Intercept | -0.34 | 0.11 | -2.97 | **0.004** | -0.56 | -0.11 |
| Child age | 0.01 | 0.00 | 4.07 | **<0.001** | 0.00 | 0.01 |
| Sex | -0.02 | 0.02 | -0.78 | 0.438 | -0.06 | 0.03 |
| Lessons – Music |  |  |  |  |  |  |
| Intercept | -0.10 | 0.07 | -1.43 | 0.157 | -0.23 | 0.04 |
| Child age | 0.00 | 0.00 | 1.94 | 0.056 | 0.00 | 0.01 |
| Sex | 0.01 | 0.01 | 0.73 | 0.465 | -0.02 | 0.04 |
| Lessons – Writing |  |  |  |  |  |  |
| Intercept | -0.12 | 0.12 | -1.06 | 0.292 | -0.35 | 0.11 |
| Child age | 0.01 | 0.00 | 2.14 | **0.035** | 0.00 | 0.01 |
| Sex | -0.00 | 0.02 | -0.10 | 0.924 | -0.05 | 0.04 |
| Lessons – Other |  |  |  |  |  |  |
| Intercept | -0.17 | 0.19 | -0.92 | 0.363 | -0.55 | 0.20 |
| Child age | 0.01 | 0.00 | 1.77 | 0.080 | -0.00 | 0.01 |
| Sex | -0.02 | 0.04 | -0.42 | 0.674 | -0.09 | 0.06 |
| Looking at pictures |  |  |  |  |  |  |
| Intercept | 0.60 | 0.53 | 1.13 | 0.262 | -0.46 | 1.67 |
| Child age | 0.01 | 0.01 | 0.54 | 0.593 | -0.01 | 0.03 |
| Sex | -0.06 | 0.10 | -0.60 | 0.554 | -0.27 | 0.14 |
| Nonphysical games |  |  |  |  |  |  |
| Intercept | 0.22 | 0.27 | 0.80 | 0.425 | -0.32 | 0.76 |
| Child age | 0.01 | 0.01 | 1.40 | 0.165 | -0.00 | 0.02 |
| Sex | -0.03 | 0.05 | -0.57 | 0.570 | -0.14 | 0.08 |
| Other unstructured activities |  |  |  |  |  |  |
| Intercept | 1.16 | 0.50 | 2.32 | **0.023** | 0.17 | 2.16 |
| Child Age | -0.01 | 0.01 | -0.75 | 0.453 | -0.03 | 0.01 |
| Sex | 0.06 | 0.10 | 0.67 | 0.507 | -0.13 | 0.26 |
| Physical games (tumbling) |  |  |  |  |  |  |
| Intercept | 1.15 | 0.50 | 2.30 | **0.024** | 0.16 | 2.15 |
| Child age | 0.00 | 0.01 | 0.37 | 0.715 | -0.01 | 0.02 |
| Sex | -0.02 | 0.10 | -0.15 | 0.879 | -0.21 | 0.18 |
| Playing instruments |  |  |  |  |  |  |
| Intercept | 0.02 | 0.11 | 0.17 | 0.867 | -0.20 | 0.23 |
| Child age | 0.00 | 0.00 | 0.71 | 0.481 | -0.00 | 0.01 |
| Sex | 0.00 | 0.02 | 0.01 | 0.994 | -0.04 | 0.04 |
| Playing outdoors (specific) |  |  |  |  |  |  |
| Intercept | 0.34 | 0.32 | 1.06 | 0.292 | -0.30 | 0.98 |
| Child age | 0.01 | 0.01 | 1.77 | 0.081 | -0.00 | 0.02 |
| Sex | -0.01 | 0.06 | -0.17 | 0.864 | -0.14 | 0.11 |
| Playing outdoors (unstructured) |  |  |  |  |  |  |
| Intercept | 1.02 | 0.29 | 3.47 | **0.001** | 0.44 | 1.61 |
| Child age | -0.01 | 0.01 | -1.18 | 0.243 | -0.02 | 0.00 |
| Sex | -0.01 | 0.06 | -0.20 | 0.846 | -0.12 | 0.10 |
| Playing with combinable objects |  |  |  |  |  |  |
| Intercept | 0.63 | 0.29 | 2.13 | **0.036** | 0.04 | 1.21 |
| Child age | 0.00 | 0.01 | 0.37 | 0.710 | -0.01 | 0.01 |
| Sex | 0.18 | 0.06 | 3.16 | **0.002** | 0.07 | 0.29 |
| Playing with free-form materials |  |  |  |  |  |  |
| Intercept | 1.00 | 0.22 | 4.66 | **<0.001** | 0.57 | 1.43 |
| Child age | -0.01 | 0.00 | -2.34 | **0.022** | -0.02 | -0.00 |
| Sex | -0.13 | 0.04 | -3.07 | **0.003** | -0.21 | -0.05 |
| Playing with loose materials |  |  |  |  |  |  |
| Intercept | 0.32 | 0.20 | 1.63 | 0.107 | -0.07 | 0.71 |
| Child age | -0.00 | 0.00 | -0.57 | 0.572 | -0.01 | 0.01 |
| Sex | 0.02 | 0.04 | 0.62 | 0.534 | -0.05 | 0.10 |
| Playing with toys |  |  |  |  |  |  |
| Intercept | 2.40 | 0.65 | 3.71 | **<0.001** | 1.12 | 3.69 |
| Child age | -0.01 | 0.01 | -0.83 | 0.412 | -0.03 | 0.01 |
| Sex | 0.03 | 0.12 | 0.21 | 0.836 | -0.22 | 0.27 |
| Pretend play |  |  |  |  |  |  |
| Intercept | 0.57 | 0.52 | 1.09 | 0.280 | -0.47 | 1.60 |
| Child age | 0.01 | 0.01 | 0.69 | 0.490 | -0.01 | 0.03 |
| Sex | -0.02 | 0.10 | -0.19 | 0.849 | -0.22 | 0.18 |
| Reading/looking at books |  |  |  |  |  |  |
| Intercept | 0.55 | 0.29 | 1.87 | 0.064 | -0.03 | 1.13 |
| Child age | 0.01 | 0.01 | 1.42 | 0.158 | -0.00 | 0.02 |
| Sex | -0.06 | 0.06 | -1.05 | 0.297 | -0.17 | 0.05 |
| Relaxation |  |  |  |  |  |  |
| Intercept | 0.22 | 0.16 | 1.38 | 0.172 | -0.10 | 0.54 |
| Child age | -0.00 | 0.00 | -0.30 | 0.764 | -0.01 | 0.00 |
| Sex | -0.03 | 0.03 | -1.05 | 0.296 | -0.09 | 0.03 |
| Unplanned activities with letters |  |  |  |  |  |  |
| Intercept | 0.43 | 0.14 | 2.99 | **0.004** | 0.14 | 0.71 |
| Child age | -0.00 | 0.00 | -1.68 | 0.097 | -0.01 | 0.00 |
| Sex | -0.03 | 0.03 | -1.15 | 0.255 | -0.09 | 0.02 |
| Unplanned activities with numbers |  |  |  |  |  |  |
| Intercept | 0.23 | 0.15 | 1.58 | 0.119 | -0.06 | 0.52 |
| Child age | -0.00 | 0.00 | -0.31 | 0.761 | -0.01 | 0.01 |
| Sex | 0.00 | 0.03 | 0.01 | 0.994 | -0.06 | 0.06 |
| Video games |  |  |  |  |  |  |
| Intercept | -0.37 | 0.21 | -1.74 | 0.086 | -0.79 | 0.05 |
| Child age | 0.01 | 0.00 | 2.49 | **0.014** | 0.00 | 0.02 |
| Sex | -0.04 | 0.04 | -1.00 | 0.321 | -0.12 | 0.04 |
| Video meeting with school |  |  |  |  |  |  |
| Intercept | -0.89 | 0.28 | -3.17 | **0.002** | -1.45 | -0.33 |
| Child age | 0.02 | 0.01 | 4.38 | **<0.001** | 0.01 | 0.03 |
| Sex | -0.05 | 0.06 | -0.98 | 0.332 | -0.16 | 0.06 |
| Video chatting with relatives/friends |  |  |  |  |  |  |
| Intercept | 0.51 | 0.21 | 2.46 | **0.016** | 0.10 | 0.92 |
| Child age | -0.00 | 0.00 | -0.32 | 0.752 | -0.01 | 0.01 |
| Sex | -0.08 | 0.04 | -2.00 | **0.049** | -0.16 | -0.00 |
| Watching TV |  |  |  |  |  |  |
| Intercept | 1.69 | 0.59 | 2.89 | **0.005** | 0.53 | 2.85 |
| Child Age | -0.01 | 0.01 | -0.75 | 0.456 | -0.03 | 0.01 |
| Sex | -0.05 | 0.11 | -0.43 | 0.671 | -0.27 | 0.18 |
| Watching videos (YouTube) |  |  |  |  |  |  |
| Intercept | 0.79 | 0.49 | 1.61 | 0.112 | -0.19 | 1.77 |
| Child age | -0.00 | 0.01 | -0.32 | 0.749 | -0.02 | 0.02 |
| Sex | -0.08 | 0.10 | -0.87 | 0.389 | -0.27 | 0.11 |
| Writing (doodling, scribbling) |  |  |  |  |  |  |
| Intercept | -0.24 | 0.23 | -1.02 | 0.313 | -0.70 | 0.23 |
| Child age | 0.01 | 0.00 | 3.30 | **0.001** | 0.01 | 0.02 |
| Sex | -0.09 | 0.05 | -1.95 | 0.054 | -0.18 | 0.00 |

*Note.* *b* = unstandardized coefficients. *SE* = standard error. *CI* = confidence interval. *LL* = lower limit. *UL* = upper limit.
